# Intermittent cafeteria diet identifies fecal microbiome changes as a predictor of spatial recognition memory impairment in female rats

**DOI:** 10.1038/s41398-020-0734-9

**Published:** 2020-01-27

**Authors:** Sarah-Jane Leigh, Nadeem O. Kaakoush, Michael J. Bertoldo, R. Frederick Westbrook, Margaret J. Morris

**Affiliations:** 1 School of Medical Sciences, UNSW, Sydney, NSW 2052 Australia; 2 Fertility and Research Centre, School of Women’s and Children’s Health, UNSW, Sydney, NSW 2052 Australia; 3 School of Psychology, UNSW, Sydney, NSW 2052 Australia

**Keywords:** Hippocampus, Molecular neuroscience, Diseases

## Abstract

Excessive consumption of diets high in saturated fat and sugar impairs short-term spatial recognition memory in both humans and rodents. Several studies have identified associations between the observed behavioral phenotype and diet-induced changes in adiposity, hippocampal gene expression of inflammatory and blood–brain barrier-related markers, and gut microbiome composition. However, the causal role of such variables in producing cognitive impairments remains unclear. As intermittent cafeteria diet access produces an intermediate phenotype, we contrasted continuous and intermittent diet access to identify specific changes in hippocampal gene expression and microbial species that underlie the cognitive impairment observed in rats fed continuous cafeteria diet. Female adult rats were fed either regular chow, continuous cafeteria diet, or intermittent cafeteria diet cycles (4 days regular chow and 3 days cafeteria) for 7 weeks (12 rats per group). Any cafeteria diet exposure affected metabolic health, hippocampal gene expression, and gut microbiota, but only continuous access impaired short-term spatial recognition memory. Multiple regression identified an operational taxonomic unit, from species *Muribaculum intestinale*, as a significant predictor of performance in the novel place recognition task. Thus, contrasting intermittent and continuous cafeteria diet exposure allowed us to identify specific changes in microbial species abundance and growth as potential underlying mechanisms relevant to diet-induced cognitive impairment.

## Introduction

There is strong evidence that diets high in saturated fat and refined carbohydrates are associated with cognitive impairments in humans^1^ and rodents^2,3^, but the mechanisms underlying this association remain unclear. Such diets compromise physiological functions across multiple organ systems, many of which could contribute to the observed cognitive impairments. Therefore, identifying key physiological disruptions that contribute to diet-induced cognitive dysfunction is essential to better understand and mitigate the effects of poor diet and obesity on cognition.

We and others have used rodent models to show that a western-style cafeteria diet, high in fat and sugar, promotes prolonged hyperphagia and increases adiposity, compromising metabolic health^4–6^. Exposure to such diets impairs performance on hippocampal-dependent tasks assessing short-term spatial recognition^2,7^ and reference^8,9^ memory, as well as disrupting multiple hippocampal molecular pathways, including pro-inflammatory signaling^7,8,10^, blood–brain barrier integrity^3,11^ and synaptic transmission^12^, and inducing fecal microbiome perturbations^13,14^. However, no one of these physiological changes has been consistently tied to the behavioral phenotype and limited studies targeting causation have been undertaken.

We have also shown that intermittent access to a western-style cafeteria diet (3 consecutive days of cafeteria diet followed by 4 consecutive days of chow diet each week for 16 weeks) resulted in similar fecal microbiome perturbations to those observed in rats continuously consuming cafeteria diet^15^ with intermediate alterations to metabolic health^16^. Of particular interest, such intermittent exposure to cafeteria diet does not impair spatial memory task performance compared with young male rats maintained on a healthy diet^17^, while still impairing metabolic health. This intermediate phenotype provides an opportunity to identify the key changes in hippocampal function and microbiome composition that are associated with diet-induced cognitive impairment, separate from more general changes produced by a cafeteria diet.

The present study aimed to determine whether specific differences in hippocampal gene expression and microbiome composition between intermittent (Cycle) and continuous cafeteria (Cafeteria) diet exposure contributed to the severity of spatial memory impairment. We chose to investigate these differences in females, as there are limited preclinical studies of the effects of obesogenic diets on cognition in female rodents despite a higher incidence of weight cycling^18^ and dieting^19^ in women.

## Materials and methods

### Ethics statement

This protocol was approved by the Animal Care and Ethics Committee of UNSW Sydney in accordance with the Australian guidelines for the use and care of animals for scientific purposes (Australian National Health and Medical Research Council).

### Subjects and diet manipulation

Thirty-six female Sprague–Dawley rats aged 4–5 months (Animal Resource Centre, Australia) were housed three per box (18–22 ^o^C; 12 h light/dark). Rats were handled daily for 1 week, while maintained on standard chow (11 kJ/g; Premium Rat and Mouse Maintenance diet; Gordon’s Specialty Stockfeeds, Australia) and water *ad libitum*.

Following acclimatization, weight-matched groups were randomly allocated to receive Chow, Cycle, or Cafeteria diet (*n* = 12 rats, *n* = 4 cages per group) *ad libitum*. The sample size was based on previous power calculations showing a minimum of ten rats is needed to detect hippocampal gene expression differences with a moderate effect size. Chow rats remained on standard chow and water. Cycle rats were exposed to 3 consecutive days of cafeteria diet, then 4 consecutive days of Chow for 7 weeks. Cafeteria rats had continuous access to cafeteria diet (full protocol in ref. ^20^) consisting of standard chow, water, and 10% sucrose solution, alongside commercially produced cakes (e.g., mud cake and jam roll), biscuits (e.g., choc-chip cookies and custard creams), and savory foods (e.g., meat pie and dim sims).

Body weight and food intake were measured twice weekly. For food intake, food items were weighed before administration and reweighed following 24 h after carefully removing all food from the cage. The amount eaten and drunk per home cage was converted to kJ using data provided by the manufacturers and the average intake per rat was calculated assuming equal intake in a cage. Body composition was analyzed at the end of week 7 by EchoMRI-900 (EchoMRI LLC, USA).

### Behavior: novel object and novel place tasks

Hippocampal-dependent spatial and perirhinal-dependent object recognition memory were assessed using the novel place recognition task (NPR) at 3 and 6 weeks, and novel object recognition task (NOR) at 4 weeks.

The apparatus was a square arena (60 cm × 60 cm × 60 cm, 40 lux). For the NOR, objects were matched on volume and color, but differed in shape and material. Object type and locations were counterbalanced across treatment groups and across time. No object was used twice for any rat. Videos were recorded to mask experimental group prior to scoring.

Two days prior to the first NPR, rats were exposed to the empty arena for 10 min each day. Both NPR and NOR tasks consisted of familiarization, retention, and test phases. During familiarization, rats were placed into the arena with two identical novel objects and allowed to explore for 5 min. They were then returned to their home cage for a 5 min retention period, while the arena and objects were cleaned with 40% ethanol.

Immediately following retention, animals underwent a 3 min test. For NOR, objects were placed in the same locations as familiarization, with one novel object and one object identical to those used previously. In NPR, the objects were identical to those shown previously, but one object was moved to a new location, while the other remained in the original location. Exploration ratio was calculated as (novel exploration time)/(novel + familiar exploration time).

### Estrous cycle monitoring

As this study examined females and diet is known to affect both the estrous cycle^21,22^ and oocyte health^23^, and there is mixed evidence for a moderating role of estrous cycle in spatial cognition^24,25^, diet- and microbiome-associated differences in estrous cycle and oocyte number were investigated. During weeks 1–2 and 6–7, daily estrous monitoring was performed by vaginal lavage. Epithelial changes were stained using 0.1% toluidine blue stain and were classified as previously reported^26^.

### Sample collection

Following 7 weeks, rats were deeply anesthetized (ketamine/xylazine 15/100 mg/kg intraperitoneally). All rats had access to their respective diets until killing (Cycle on cafeteria diet). Body weight, naso-anal length, girth, and blood glucose were measured following induction of anesthesia. Blood was obtained by cardiac puncture and rats were decapitated. Plasma was stored at −80 ^o^C for subsequent determination of leptin, insulin, and triglyceride content.

The dorsal hippocampus (within a coronal block defined by the rostro-caudal limits of the Circle of Willis) was rapidly dissected and collected. Retroperitoneal white adipose tissue and liver were dissected and weighed. One fecal pellet was removed from the distal colon. Hippocampus and feces were snap frozen in liquid nitrogen and stored at −80 ^o^C for analysis. Ovaries were dissected and oocytes recovered and counted.

### Plasma hormone and triglyceride measurements

Plasma leptin and insulin concentrations were obtained using commercial kits according to manufacturer’s instructions (CAT#90040 and CAT#90060, CrystalChem, Inc., USA).

Plasma triglyceride content was measured spectrophotometrically using triglyceride reagent (Roche Diagnostics Australia Pty, Ltd, Australia) at 37 °C alongside a standard curve generated from glycerol standard (G7793-5ML, Sigma-Aldrich Pty, Ltd, Australia).

### Hippocampal gene expression

RNA was extracted from dorsal hippocampus using a TRI Reagent protocol (Sigma-Aldrich Pty, Ltd, Australia). Following DNAse I treatment (Catalog# 42885; Merck, Australia), 1.5 μg of RNA were reverse transcribed to produce cDNA (High Capacity Reverse Transcriptase Kit; Thermo Fisher Scientific, USA). Gene expression was assessed using Taqman inventoried gene expression assays (Life Technologies Australia Pty, Ltd, Australia; details in Supplementary Table 2). Genes of interest were normalized against the geometric mean of the two most stable housekeeping genes (*Ywhaz* and *Hprt1*) identified by Normfinder code in R^27^. Analysis of relative gene expression was performed using the ∆∆CT method normalized to an independent calibrator^28^.

### Statistical analyses

Data were analyzed using one-way analysis of variance (ANOVA) or mixed two-way ANOVA (for measures over time) with post-hoc Tukey’s comparisons. Where data violated homoscedasticity of variance, log transformations were employed. Ordinal variables were analyzed using an independent-samples Jonckheere–Terpstra test for ordered alternatives followed by non-parametric Bonferroni–Dunn post-hoc testing. All post-hoc comparisons are presented only in the associated figures and tables when *p* < 0.05. Pearson’s correlations were performed to identify key variables of interest associated with NPR performance, which was followed up using simultaneous multiple regression to identify key predictors. All analyses were completed using IBM SPSS Statistics 23 (Australia). One Cafeteria rat was excluded due to poor health (hepatomegaly).

### Fecal DNA extraction, microbiome community sequencing, and statistical analyses

DNA extraction was performed using the PowerFecal DNA Isolation Kit (MoBio Laboratories, USA). Microbial community composition was assessed by Illumina amplicon sequencing (2 × 250 bp MiSeq chemistry, V4 region, 515F-806R primer pair) using a standard protocol. Sequence data were analyzed using MOTHUR^29^, using modified commands from MiSeq SOP^30^, including alignment with the SILVA database, singleton removal, chimera checking with UCHIME, and classification against the latest RDP training set. Sequence data (*n* = 8346 total clean reads/sample) did not undergo rarefaction.

Operational taxonomic unit (OTU) correlations and LefSe analyses were completed using Calypso^31^, where multiple testing was corrected using the Benjamini–Hochberg false discovery rate (FDR). Alpha diversity metrics were obtained from Calypso and analyses were completed using SPSS, whereas FDR-corrected DESeq2 was performed using R, using the Phyloseq^32^ package for the negative binomial Walk’s test in DESeq2^33^. OTU abundances were analyzed using SPSS with Kruskal–Wallis tests when necessary, followed by non-parametric Bonferroni–Dunn post-hoc testing where appropriate. OTUs of interest were identified using SINA Aligner^34^ and BlastN.

Distanced-based linear modeling (dbLM), permutational ANOVA (PERMANOVA), non-metric multidimensional scaling (nMDS), and canonical analysis of principal coordinates were completed using Primer (Primer-E Ltd, Plymouth, UK^35^). All primer analyses utilized a Bray–Curtis similarity matrix constructed at the OTU level. Marginal dbLM interrogates the unique contribution of each predictor variable to the variance in the Bray–Curtis similarity matrix.

Phylogenetic Investigation of Communities by Reconstruction of Unobserved States (PICRUSt) was performed using Galaxy web, to predict putative functions (through metagenomic prediction) from the 16S OTU data using Greengenes 13.5 for taxonomic classification^36^. Pathway counts were compared across groups using FDR-corrected Kruskal–Wallis tests followed by non-parametric Bonferroni–Dunn post-hoc testing, where appropriate.

## Results

### Continuous cafeteria diet impairs metabolic health more than diet cycling

Access to cafeteria diet consistently elevated daily energy intake: Cafeteria and Cycle rats consumed on average 3.8- and 1.8-fold more energy than chow controls (41,920 kJ/rat, 19,980 kJ/rat, and 11,133 kJ/rat total energy, respectively). Based on food-intake measurements, Cafeteria rats consumed 58% carbohydrates, 8% protein, and 34% fat as energy; similarly, Cycle rats consumed 56% carbohydrates, 8% protein, and 35% fat while on cafeteria diet. Chow diet comprised 65% carbohydrate, 22% protein, and 13% fat.

Absolute protein intake was slightly elevated in Cafeteria rats relative to Chow. Cycle rats ate comparable protein to Cafeteria rats, while consuming cafeteria diet; however, they consumed slightly less protein than Chow rats overall (Supplementary Fig. 1C; total protein: Chow: 2473 kJ/rat, Cycle: 2036 kJ/rat, and Cafeteria: 3092 kJ/rat). The intake of carbohydrate and fat (Supplementary Fig. 1D and E) when Cycle rats were exposed to the cafeteria diet were comparable to those by Cafeteria rats and were higher than Chow intakes when summed over the study (total carbohydrate: Chow: 7418 kJ/rat, Cycle: 11,524 kJ/rat, Cafeteria: 24,351 kJ/rat; total fat: Chow:1538 kJ/rat, Cycle: 6192 kJ/rat, Cafeteria: 14,184 kJ/rat).

Body weight gain over the study was significantly different between groups (F(2,32) = 49.62, *p* < 0.001; Supplementary Fig. 1F for body weight over time) such that weight gain was greater in Cafeteria rats than Chow and Cycle rats. There was no significant difference between Chow and Cycle rats, in line with our previous work in male rats^37^.

Continuous cafeteria diet increased body weight, fat mass, liver weight, and blood glucose, as well as plasma insulin, leptin, and triglyceride concentrations, relative to Chow rats with less marked effects in Cycle rats. Although lean mass did not differ, fat mass and leptin concentrations were significantly different among all groups, increasing with diet exposure. Oocyte number was reduced in Cafeteria rats relative to Chow controls (Table 1).

**Table 1:** Body composition, tissue collection, and plasma measures.

	Chow	Cycle	Cafeteria	Diet effect
EchoMRI (3 days prior to kill)				
Fat mass (g)	46.8 ± 4.1	73.1 ± 6.8^a^	126.1 ± 9.6^a,b^	*p* < 0.001
Fat percentage (%)	14.2 ± 1.1	20.7 ± 1.4^a^	29.5 ± 1.4^a,b^	*p* < 0.001
Lean mass (g)	262.6 ± 6.5	256.2 ± 5.4	277.0 ± 7.4	–
Tissue collection				
Final body weight (g)	329.9 ± 7.9	367.0 ± 12.0	433.4 ± 17.3^a,b^	*p* < 0.001
Liver weight (g)	9.95 ± 0.35	11.96 ± 0.54^a^	12.68 ± 0.49^a^	*p* = 0.001
Retroperitoneal fat pad weight (g)	4.81 ± 0.45	9.40 ± 1.14^a^	13.15 ± 0.71^a,b^	*p* < 0.001
Ovary weight (mg)	46.0 ± 2.1	44.7 ± 1.93	50.2 ± 2.4	–
Total oocytes/female	20.5 ± 1.3	17.1 ± 1.5	15.1 ± 1.8^a^	*p* = 0.024
Blood/plasma measures				
Blood glucose (mmol/L)	7.7 ± 0.3	8.3 ± 0.3	9.3 ± 0.5^a^	*p* = 0.014
Plasma insulin (ng/mL)	0.19 ± 0.05	0.91 ± 0.20^a^	1.08 ± 0.22^a^	*p* = 0.002
Plasma leptin (ng/mL)	5.69 ± 0.68	11.35 ± 1.28^a^	17.38 ± 1.67^a,b^	*p* < 0.001
Plasma triglycerides (mmol/L)	1.06 ± 0.08	2.30 ± 0.34^a^	3.48 ± 0.49^a^	*p* = 0.001

Results expressed as mean ± SEM; *n* = 11–12; data were analyzed by one-way ANOVA followed by Tukey-adjusted post-hoc testing (^a^*p* < 0.05 relative to Chow, ^b^*p* < 0.05 relative to Cycle). Total oocytes per female were analyzed using an independent-samples Jonckheere–Terpstra test for ordered alternatives followed by non-parametric Bonferroni–Dunn post-hoc testing (^a^*p* < 0.05 relative to Chow).

### Intermittent cafeteria diet spares cognition and increases the expression of some hippocampal pro-inflammatory genes

Short-term spatial and object memory recognition were assessed using NPR and NOR tasks, respectively; when assessed with a short retention period (5 min), the NPR is predominantly hippocampal-dependent, whereas the NOR relies on an intact perirhinal cortex^38,39^. Previously, we have shown that NPR, but not NOR, is impaired by exposure to cafeteria diet in rats^2,7^.

**Fig. 1:**
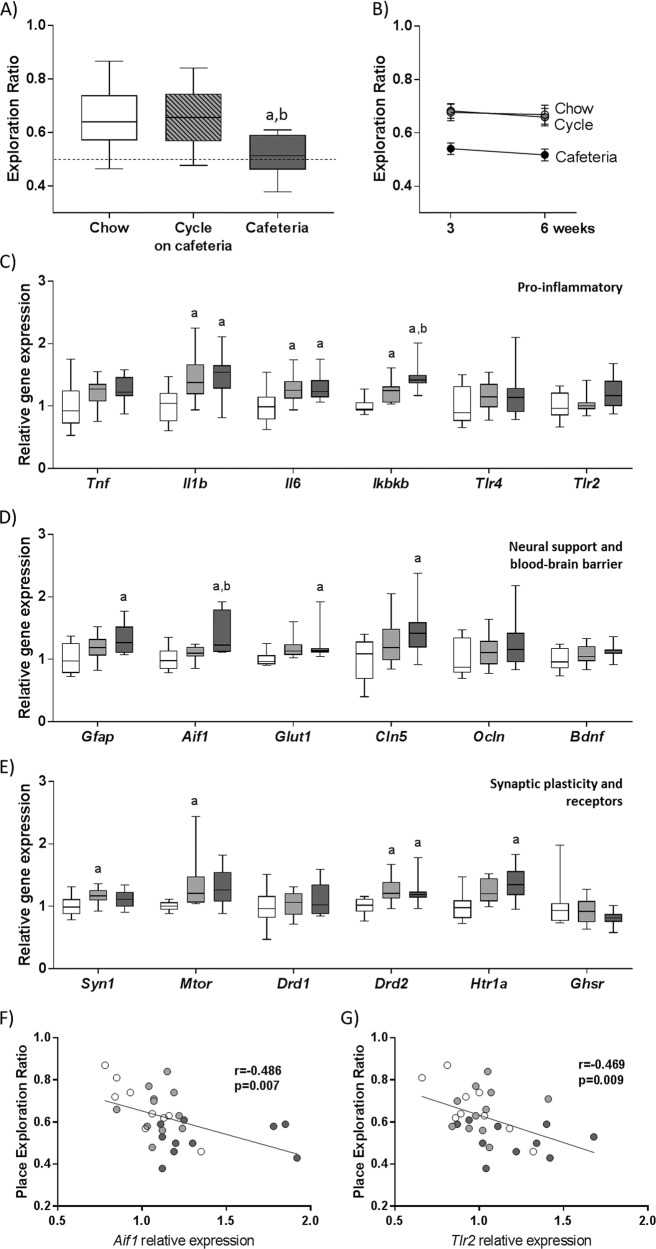
Intermittent cafeteria diet exposure spares spatial recognition memory while increasing expression of some hippocampal pro-inflammatory genes. **a** Novel place recognition memory task performance at 6 weeks of diet exposure. **b** Novel place recognition memory task performance over the study. **c** Expression of pro-inflammatory genes in the dorsal hippocampus. **d** Expression of glial and blood–brain barrier-related genes in the dorsal hippocampus. **e** Expression of neuroplasticity-related and receptor genes in the dorsal hippocampus. Data are expressed as box-and-whisker plots (min, IQR, max); *n* = 10–12; data were analyzed by one-way ANOVA followed by Tukey-adjusted post-hoc comparisons (^a^*p* < 0.05 relative to Chow, ^b^*p* < 0.05 relative to Cycle). **f** Correlation between spatial recognition memory and hippocampal *Aif1* gene expression. **g** Correlation between spatial recognition memory and hippocampal *Tlr2* gene expression. Data are expressed as scatterplots of individual values; *n* = 9–12; data were analyzed by Pearson’s correlations. *Aif1* allograft inflammatory factor 1, *Bdnf* brain-derived neurotrophic factor, *Cln5* Claudin 5, *Drd1* dopamine receptor D1, *Drd2* dopamine receptor D2, *Gfap* glial fibrillary acidic protein, *Ghsr* growth hormone secretagogue receptor, *Glut1* glucose transporter 1, *Htr1a* serotonin receptor 1a, *Ikbkb* inhibitor of nuclear factor κB kinase subunit β, *Il1b* interleukin-1β, *Il6* interleukin-6, *Mtor* mammalian target of rapamycin, *Ocln* occludin, *Syn1* synapsin 1, *Tlr2* Toll-like receptor 2, *Tlr4* Toll-like receptor 4, *Tnf* tumor necrosis factor-α.

Following 3 weeks of diet exposure, NPR memory was significantly affected by diet (F(2,32) = 9.08, *p* < 0.001; Fig. 1b and Supplementary Fig. 2A, B). Although both Chow and Cycle rats performed significantly better than Cafeteria, there was no difference between Chow and Cycle rats. By contrast, there were no significant group differences for NOR at 4 weeks (F(2,32) < 1; Supplementary Fig. 2E, F), consistent with previous work^2,7^.

The group differences in NPR performance present at 3 weeks remained significant at 6 weeks (F(2,32) = 7.57, *p* = 0.022; Fig. 1a, b), when Cycle rats had experienced 19–20 days total cafeteria diet, matching exposure of Cafeteria rats at the 3-week time point. Tested 4 days later, although Cycle rats were consuming chow, the overall effect of diet remained significant (F(2,31) = 5.71, *p* = 0.008; Supplementary Fig. 2C, D) such that Cafeteria rats were significantly impaired relative to Cycle rats. There was no significant difference between Chow and Cycle rat performance on the NPR task. Exploration times were not significantly different between groups for any of the tasks (Supplementary Fig. 1).

As there is some evidence that spatial cognition may vary with estrous cycle in female rodents^24,25^, we assessed estrous cycle after the final NPR. No relationship was observed with NPR performance, although this was not explicitly manipulated, as we did not want to introduce variability in dietary exposure by matching estrous cycle across groups.

Hippocampal gene expression was assessed to determine whether diet affected markers of pro-inflammatory signaling, blood–brain barrier integrity, and synaptic function (Fig. 1c-e and Supplementary Table 1 for gene names). Overall, these results indicate that although any exposure to cafeteria diet increases hippocampal cytokine expression, increased markers of astroglial and microglial proliferation, downstream pro-inflammatory signaling, and changes to blood–brain barrier integrity were unique to continuous cafeteria diet exposure. Specifically, any cafeteria diet exposure increased expression of *Il1b* (F(2,29) = 6.13, *p* = 0.006), *Il6* (F(2,29) = 5.17, *p* = 0.012), *Ikbkb* (F(2,29) = 17.05, *p* < 0.001), and *Drd2* (F(2,29) = 6.091, *p* = 0.006), while continuous access uniquely elevated expression of *Gfap* (F(2,29) = 4.948, *p* = 0.014), *Glut1* (F(2,29) = 3.827, *p* = 0.036), *Cln5* (F(2,29 = 3.846, *p* = 0.033), and *Htr1a* (F(2,29) = 6.64, *p* = 0.004) relative to Chow. *Aif1* (gene for Iba1, a marker of microglial proliferation; F(2,29) = 8.33, *p* = 0.001) was significantly elevated in Cafeteria rats relative to both Chow and Cycle groups. Interestingly, *Syn1* (F(2,29) = 3.886, *p* = 0.032), *Mtor* (F(2,29) = 4.214, *p* = 0.025), and *Mc4r* (F(2,29) = 5.98, *p* = 0.007; Supplementary Fig. 3C) were elevated in Cycle rats, suggesting differences in synaptic plasticity and energy homeostasis signaling exclusively in this group. *Lepr* (F(2,29) = 1.83, 0.18; Supplementary Fig. 3A), *Insr* (F(2,29) = 1.82, *p* = 0.18; Supplementary Fig. 3B), and *Glut3* (F(2,29) = 1.7, *p* = 0.21; Supplementary Fig. 3D) were also assessed to determine whether there were any group differences in metabolic signaling, but did not show statistically significant differences. *Aif1* and *Tlr2* were the only genes significantly associated with spatial recognition memory (Fig. 1f and g, and Supplementary Table 2), implying that bacterial lipoprotein content and microglial proliferation may be involved in diet-induced cognitive impairment.

### Intermittent cafeteria diet exposure does not affect α-diversity or predicted bacterial function, but alters microbiome composition

**Fig. 2:**
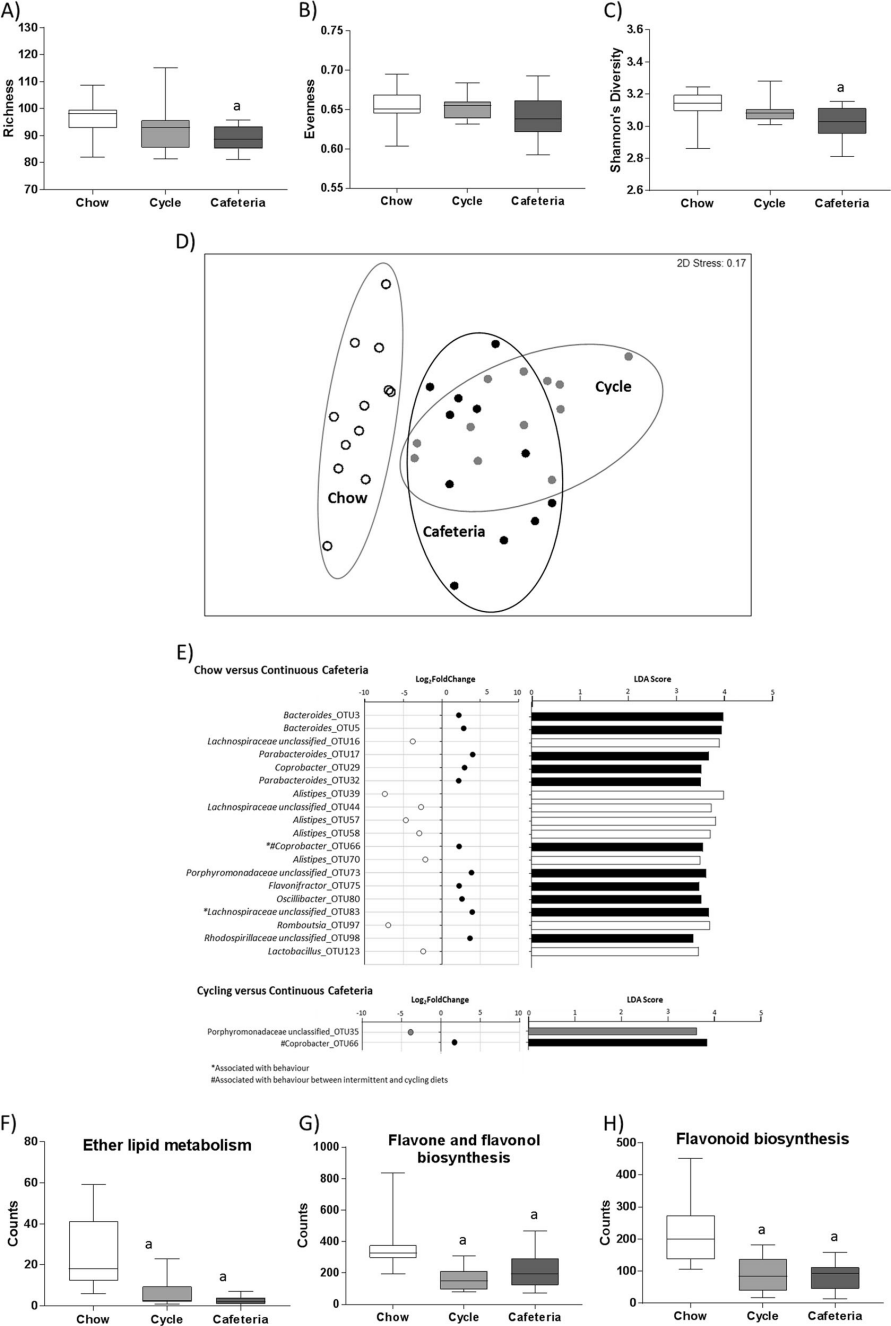
Any cafeteria diet exposure affects gut microbiome composition and alters predicted bacterial metabolic pathways. **a** Microbial species richness, **b** microbial species evenness, and **c** Shannon’s diversity following 7 weeks of diet exposure. Data are expressed as box-and-whisker plots (min, IQR, max); *n* = 11–12; data were analyzed by one-way ANOVA followed by Tukey-adjusted post-hoc comparisons (^a^*p* < 0.05 relative to Chow). **d** Non-metric multidimensional scaling (using Bray–Curtis similarity scores) on fecal microbiome samples; *n* = 11–12. **e** Operational taxonomic units (OTUs) differentially expressed between Chow, Cycle, and Cafeteria groups. Data are expressed using both DeSeq2 (log(Fold change), FDR-adjusted *p*-value < 0.05), and LefSe (LDA score > 2, FDR-adjusted *p* < 0.05) scores. The color of the circle/bar denotes the group the OTU is enriched in (open: Chow, gray: Cycle, black: Cafeteria); *n* = 11–12. **f** Ether lipid metabolism, **g** flavone and flavonol biosynthesis, and **h** flavonoid biosynthesis predicted using PICRUSt from fecal microbiome data at 7 weeks. Data are expressed as box-and-whisker plots (min, IQR, max); *n* = 11–12; data were analyzed by one-way ANOVA (FDR-adjusted overall *p*-value to account for multiple relevant pathways included in the analysis) followed by Tukey-adjusted post-hoc comparisons (^a^*p* < 0.05 relative to Chow, ^b^*p* < 0.05 relative to Cycle).

Microbial species diversity was assessed using richness, evenness, and Shannon’s diversity at 7 weeks. Both richness (F(2,33) = 3.949, *p* = 0.029; Fig. 2a) and Shannon’s diversity (F(2,33) = 4.31, *p* = 0.022; Fig. 2c) were significantly affected by diet overall, and the Cafeteria group showed reduced values relative to chow-fed controls. Evenness (Fig. 2b) was not significantly changed by diet. No significant effect of cage was observed.

PERMANOVA (999 permutations) at 7 weeks revealed significant differences in microbiome composition by diet (Pseudo-F(2,24) = 3.18, *p* = 0.006) and cage (Pseudo-F(3,24) = 1.51, *p* = 0.001), and the nMDS plot illustrating the similarity between groups is presented in Fig. 2d. Canoncal analysis of principal coordinates revealed that although Cycle microbiome overlapped with both Chow and Cafeteria groups, it was more closely aligned with the Cafeteria microbiome composition: when combined Cycle and Cafeteria were compared with Chow, there was 0% mis-classification error, whereas this was 14.3% for combined Cycle and Chow vs. Cafeteria. Overall, there were no significant differences in dispersion between groups (F(2,33) = 1.55, *p* = 0.235).

Several relative OTU abundances were selectively enriched in particular diet groups, using both LefSe and DeSeq2 analyses (DeSeq2: FDR-adjusted *p* < 0.05; LefSe: LDA score > 2, *p* < 0.05; Fig. 2e). When comparing Chow and Cafeteria, 16 OTUs were enriched in cafeteria diet, whereas 15 OTUs were enriched in chow-fed controls, and similar differences were observed between Chow and Cycle rats. Between Cycle and Cafeteria groups, only two OTUs were differentially enriched: *Porphyromonadaceae unclassified_*OTU35 was enriched in Cycle rats, whereas *probacter_*OTU66 was relatively enriched in the Cafeteria group.

Predicted bacterial function was assessed using PICRUSt analysis of 16S sequence data. Following FDR correction for multiple comparisons on relevant bacterial and metabolic KEGG (Kyoto Encyclopedia of Genes and Genomes) pathways, ether lipid metabolism (H(2) = 19.99, *p* = 0.005), flavone and flavonol biosynthesis (H(2) = 15.42, *p* = 0.005), and flavonoid biosynthesis (H(2) = 16.90, *p* = 0.001) were all significantly reduced by any cafeteria diet exposure but none of these changes were associated with NPR performance.

### Changes in global microbiome composition are associated with place task performance

Several variables were significantly associated with overall microbiome composition when their unique variance was considered through dbLM (Table 2), including measures of adiposity as well as hippocampal *Ikbkb* and *Il1b*. Of note, NPR performance at 6 weeks was a significant predictor of global microbiome composition (Pseudo-F = 1.89, *p* = 0.022, *R*^2^ = 0.054).

**Table 2:** Correlations between global microbiome composition and variables of interest.

Variable	SS	Pseudo-F	*P*-value	R^2^	Residual DF
Fat mass	4061.7	2.716	**0.004**	0.0761	33
Blood glucose	3679.1	2.442	**0.003**	0.0689	33
Leptin	4818.1	3.272	**0.002**	0.0902	33
Insulin	3037.7	1.990	**0.022**	0.0569	33
Plasma triglycerides	3305.9	2.178	**0.015**	0.0619	33
Liver weight	4201.2	2.818	**0.002**	0.0787	33
Heart weight	1970.1	1.264	0.175	0.0369	33
Average oocytes per female	2539.5	1.648	0.056	0.0476	33
Hippocampal *Aif1*	1875.6	1.176	0.287	0.0377	30
Hippocampal *Il1b*	2711.6	1.730	**0.050**	0.0545	30
Hippocampal *Il6*	2394.6	1.518	0.099	0.0482	30
Hippocampal *Ikbkb*	2958.8	1.898	**0.033**	0.0595	30
Hippocampal *Tlr4*	1774.8	1.110	0.335	0.0357	30
Hippocampal *Glut1*	2120.9	1.337	0.178	0.0427	30
Hippocampal *Cln5*	1896.3	1.189	0.302	0.0381	30
Hippocampal *Syn1*	2316.7	1.466	0.120	0.0466	30
Place exploration ratio	2895.4	1.892	**0.022**	0.0542	33

Marginal distance-based linear modeling (dbLM) was performed using a Bray–Curtis similarity matrix of the global microbiome composition at the operational taxonomic unit level with variables of interest. Marginal dbLM interrogates the unique contribution of each predictor variable to the variance explained in the Bray–Curtis similarity matrix Pseudo-F and *p*-values were obtained using 999 permutations and bold values indicate *p*-values ≤ 0.05; *N* = 32–36. All variable were tested, only those with a *p*-value < 0.4 are presented. *Aif1* allograft inflammatory factor 1, *Cln5* Claudin 5, *Glut1* glucose transporter 1, *Ikbkb* inhibitor of nuclear factor κB kinase subunit-β, *Il1b* interleukin-1β, *Il6* interleukin-6, *Syn1* synapsin 1, *Tlr4* Toll-like receptor 4.

### *Coprobacter_*OTU66 and body length are significant predictors of place task performance

**Fig. 3:**
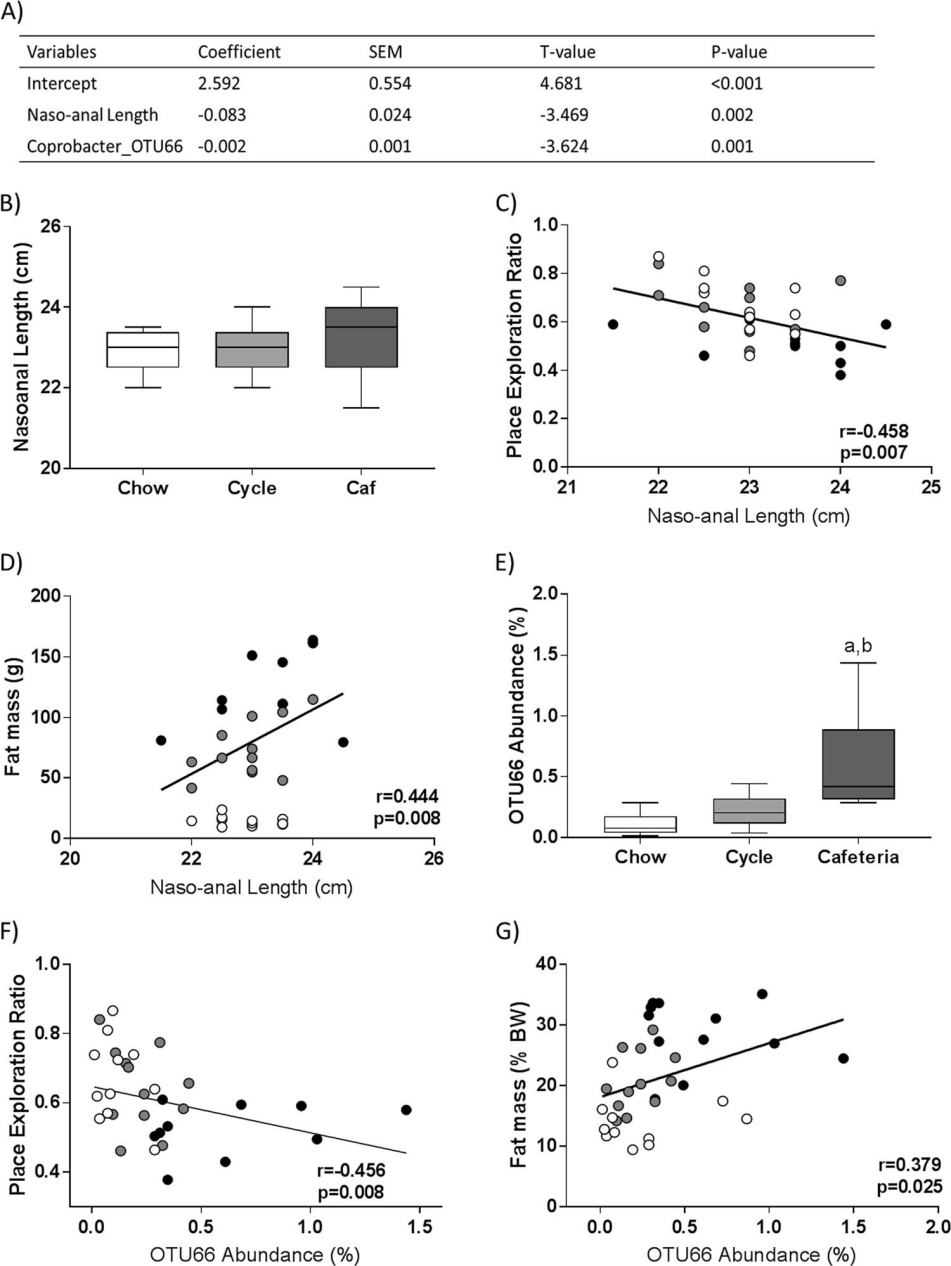
Naso-anal length and *Coprobacter*_OTU66 are significant predictors of place task performance in a multiple regression model. **a** Final multiple regression model (F(2,30) = 12.28, *p* < 0.001) predicting 41.3% of the variance in place task performance following the removal of nonsignificant predictors. **b** Naso-anal length at 7 weeks and its associations with (**c**) spatial recognition memory and (**d**) fat mass. **e** Relative abundance of Coprobacter_OTU66 at 7 weeks and its associations with (**f**) spatial recognition memory and (**g**) fat mass. Group data are expressed as box-and-whisker plots (min, IQR, max); *n* = 11–12; data were analyzed by one-way ANOVA followed by Tukey-adjusted post-hoc comparisons (^a^*p* < 0.05 relative to Chow, ^b^*p* < 0.005 relative to Cycle). Associational data are expressed as scatterplots of individual values; *n* = 9–12; data were analyzed by Pearson’s correlations.

Several variables of interest were significantly associated with spatial recognition memory using Pearson’s correlations (Supplementary Table 2). Although many differentially abundant OTUs correlated with NPR performance, only the association with *Coprobacter_*OTU66 (*r* = −0.456, *p* = 0.008; Fig. 3f) was not dependent on large outliers. Of note, this association was statistically significant in Chow rats only (*r* = −0.899, *p* < 0.001) when investigated within each diet group. *Coprobacter*_OTU66 was also significantly associated with girth, fat mass, and a number of hippocampal pro-inflammatory genes (Supplementary Table 2). These variables were then entered into a sequential multiple regression, to determine which significantly predicted NPR performance while accounting for other variables. The final model, presented in Fig. 3a, found that *Coprobacter*_OTU66 and naso-anal length significantly predict 41.4% of the variance in NPR (F(2,30) = 12.28, *p* < 0.001). Naso-anal length did not differ significantly between groups (Fig. 3b), although its strong positive association with fat mass (*r* = 0.444, *p* = 0.008; Fig. 3d) and other measures of adiposity indicate that this may be an indirect measure of diet impact.

*Coprobacter*_OTU66 was enriched in Cafeteria rats relative to Chow, as well as in Cafeteria relative to Cycle rats (Fig. 3e), and positively associated with fat mass (*r* = 0.379, *p* = 0.025; Fig. 3g). SINA Aligner analysis identified one uncultured bacterium from the family *Muribaculaceae* (Accession number EU451781 ENA) isolated from mouse cecum^40^ that shared 90.5% sequence identity with *Coprobacter*_OTU66. BLAST analysis showed *Coprobacter_*OTU66 shared 90% sequence identify with *Muribaculum intestinale* strain YL27.

## Discussion

In contrast to continuous access to cafeteria diet, we found that intermittent diet exposure in females (Cycle group) generated an intermediate phenotype, with moderate increases in energy intake and adiposity, perturbed fecal microbiome composition, but no cognitive impairment. This pattern of access to a cafeteria diet has been shown to spare spatial recognition in male rats^17^, which is interesting, as poor diet has been shown to impair cognition when presented in a daily limited access model^41^ and when excess energy intake is prevented^10^. Such intermittent access to cafeteria may spare cognition through the hypophagia exhibited during access to healthy chow, as both chronic^42^ and intermittent^43^ caloric restriction have been shown to improve cognition in mice.

Although Cycle rats developed a similar metabolic phenotype to Cafeteria rats, they exhibited lower relative fat mass and plasma leptin concentrations, which could explain the apparent protective effects of intermittent cafeteria diet on cognition. Of note, the plasma measures were conducted while animals were unfasted, due to previous work showing that diet-induced hypothalamic neuroinflammation can be reversed with overnight fasting^44^, and this could potentially mask differences in metabolic function between Cycle and Cafeteria rats. However, we have previously shown that diet-induced cognitive impairments occur without increased body weight^7,10^. Furthermore, although changes in hippocampal pro-inflammatory gene expression (*Tlr2* and *Aif1*) were associated with spatial recognition memory independently, as seen in other rodent studies^2,11,45^, these were no longer significant predictors of the behavioral phenotype when multiple anthropometric and microbiome predictors of NPR performance were considered, most likely due to issues of multicollinearity. These findings indicate that although increased adiposity and hippocampal gene expression may contribute to cognitive impairments observed in obesity^46–48^, these changes are highly related and are therefore difficult to disentangle. Overall, it appears that different schedules of cafeteria diet induce incremental increases in hippocampal dysfunction and inflammation, and metabolic impairment, and it may be that some or all these changes must reach a threshold before diet-induced cognitive impairment is observed.

Cycle rats exhibited an intermediate microbiome composition that was significantly different from both Chow and Cafeteria groups, although exhibiting significant overlap with the Cafeteria group. This is similar to previous observations in mice exposed to 4-week cycles between chow and high-fat diet^49^ or chronic caloric restriction while on high-fat diet^50^, as well as our previous work with male rats exposed to long-term cafeteria diet cycling^15^. Although overall microbiome composition was altered, diet cycling protected female rats from the reductions in measures of α-diversity typically seen with extended exposure to a cafeteria diet^15,51^. Reduced α-diversity measures have been consistently reported in other diet-induced obesity models^52,53^, and following 4-week periods of cycling between healthy and high-fat diets^49^. Our previous diet cycling work in male rats showed comparable reductions in richness between rats exposed to cycling and continuous cafeteria diet^15^, although this may have been due to either longer diet exposure of 16 weeks.

Naso-anal length and *Coprobacter*_OTU66 were identified as significant predictors of place task performance through multiple regression modeling. Naso-anal length is typically increased with cafeteria diet in rodents^17,54^ and, although it was not significantly increased with diet in this study, it was highly correlated with measures of adiposity, which is known to be negatively associated with cognition in both humans^55,56^ and rodents^57^. This relationship between spatial recognition and naso-anal length is likely specific to rodents, who continue to grow linearly across their lifespan. When naso-anal length was removed from the regression analysis, lean mass became a significant predictor of place task performance. Lean mass is also increased with an energy dense diet in rodents, most likely due to diet-induced growth as well as increased organ weights. Contrastingly, lean mass is protective against cognitive in aging humans^58,59^ and is positively associated with hippocampal-dependent lure discrimination in healthy young adults^60^.

*Coprobacter*_OTU66 was putatively identified as *M. intestinale* strain YL27 and has only been recently sequenced^61^. In mice, this strain has been associated with a healthy phenotype^62^ and low-fat diet consumption^61^. Future studies manipulating this strain in rats may provide further insight into its role in diet-induced obesity and cognitive dysfunction.

It is important to note that we failed to detect any association between estrous stage and spatial task performance, but did observe a lengthening of proestrous and a reduction in diestrous stage 1/2 of the Cafeteria rats, in line with other rodent work^21,63^. We subsequently investigated the rats’ ovaries and found that oocyte number was reduced by continuous cafeteria diet exposure. Similarly, others have shown that Ob/ob mice exhibit reduced oocyte number^64^ and high-fat fed mice are less fertile due to increased oxidative stress-induced apoptosis and impaired oocyte maturation^65^.

In summary, our model of intermittent access to cafeteria diet identified *OTU66_Coprobacter* (high similarity to *M. intestinale* strain YL27) and naso-anal length, an indirect measure of adiposity and diet-induced growth, as predictive of differences in spatial recognition memory observed with differing schedules of cafeteria diet access in female rats. This approach appears useful for identifying microbiome composition differences that are relevant to phenotypic differences, rather than overall diet effects, and may prove useful in future studies investigating microbiome differences associated with cafeteria diet exposure.

## Supplementary information

Supplementary Figures and Tables

## Data Availability

The study metadata and sequence data are available in the European Nucleotide Archive under accession number PRJEB32323. All other data will be made available upon from the corresponding author on reasonable request.

## References

[1] O’Brien PD, Hinder LM, Callaghan BC, Feldman EL (2017). Neurological consequences of obesity. Lancet Neurol..

[2] Beilharz JE, Maniam J, Morris MJ (2016). Short-term exposure to a diet high in fat and sugar, or liquid sugar, selectively impairs hippocampal-dependent memory, with differential impacts on inflammation. Behav. Brain Res..

[3] Kanoski SE, Zhang Y, Zheng W, Davidson TL (2010). The effects of a high-energy diet on hippocampal function and blood-brain barrier integrity in the rat. J. Alzheimers Dis..

[4] South T, Westbrook F, Morris MJ (2012). Neurological and stress related effects of shifting obese rats from a palatable diet to chow and lean rats from chow to a palatable diet. Physiol. Behav..

[5] Gomez-Smith M (2016). A physiological characterization of the Cafeteria diet model of metabolic syndrome in the rat. Physiol. Behav..

[6] Higa TS, Spinola AV, Fonseca-Alaniz MH, Evangelista FS (2014). Comparison between cafeteria and high-fat diets in the induction of metabolic dysfunction in mice. Int J. Physiol. Pathophysiol. Pharm..

[7] Beilharz JE, Maniam J, Morris MJ (2014). Short exposure to a diet rich in both fat and sugar or sugar alone impairs place, but not object recognition memory in rats. Brain Behav. Immun..

[8] Lewis AR, Singh S, Youssef FF (2019). Cafeteria-diet induced obesity results in impaired cognitive functioning in a rodent model. Heliyon.

[9] Ferreira A, Castro JP, Andrade JP, Dulce Madeira M, Cardoso A (2018). Cafeteria-diet effects on cognitive functions, anxiety, fear response and neurogenesis in the juvenile rat. Neurobiol. Learn Mem..

[10] Beilharz JE, Kaakoush NO, Maniam J, Morris MJ (2016). The effect of short-term exposure to energy-matched diets enriched in fat or sugar on memory, gut microbiota and markers of brain inflammation and plasticity. Brain Behav. Immun..

[11] Hargrave SL, Davidson TL, Zheng W, Kinzig KP (2016). Western diets induce blood-brain barrier leakage and alter spatial strategies in rats. Behav. Neurosci..

[12] Stranahan AM (2008). Diet-induced insulin resistance impairs hippocampal synaptic plasticity and cognition in middle-aged rats. Hippocampus.

[13] Beilharz JE, Kaakoush NO, Maniam J, Morris MJ (2018). Cafeteria diet and probiotic therapy: cross talk among memory, neuroplasticity, serotonin receptors and gut microbiota in the rat. Mol. Psychol..

[14] Del Bas JM (2018). Alterations in gut microbiota associated with a cafeteria diet and the physiological consequences in the host. Int. J. Obes. (Lond.).

[15] Kaakoush, N. O. et al. Alternating or continuous exposure to cafeteria diet leads to similar shifts in gut microbiota compared to chow diet. *Mol. Nutr. Food Res.* **61**, 1500815 (2017). 10.1002/mnfr.201500815 26767716

[16] Martire SI, Westbrook RF, Morris MJ (2015). Effects of long-term cycling between palatable cafeteria diet and regular chow on intake, eating patterns, and response to saccharin and sucrose. Physiol. Behav..

[17] Kendig MD, Westbrook RF, Morris MJ (2019). Patterns of access to cafeteria-style diet determines fat mass and degree of spatial memory impairments in rats. Sci. Rep..

[18] George VA, Johnson P (2001). Weight loss behaviors and smoking in college students of diverse ethnicity. Am. J. Health Behav..

[19] Paeratakul S, York-Crowe EE, Williamson DA, Ryan DH, Bray GA (2002). Americans on diet: results from the 1994-1996 Continuing Survey of Food Intakes by Individuals. J. Am. Diet. Assoc..

[20] Leigh SJ, Kendig MD, Morris MJ (2019). Palatable western-style cafeteria diet as a reliable method for modeling diet-induced obesity in rodents. JoVE.

[21] Balasubramanian P (2012). High fat diet affects reproductive functions in female diet-induced obese and dietary resistant rats. J. Neuroendocrinol..

[22] Leigh AJ, Stock MJ, Lacey JH, Wilson CA (1998). Diet-induced loss of cyclic ovarian function at normal body weight in a rodent model for bulimia nervosa. J. Reprod. Fertil..

[23] Snider AP, Wood JR (2019). Obesity induces ovarian inflammation and reduces oocyte quality. Reproduction (Camb., Engl.).

[24] Abbott KN, Morris MJ, Westbrook RF, Reichelt AC (2016). Sex-specific effects of daily exposure to sucrose on spatial memory performance in male and female rats, and implications for estrous cycle stage. Physiol. Behav..

[25] Sutcliffe JS, Marshall KM, Neill JC (2007). Influence of gender on working and spatial memory in the novel object recognition task in the rat. Behav. Brain Res..

[26] Cora MC, Kooistra L, Travlos G (2015). Vaginal cytology of the laboratory rat and mouse: review and criteria for the staging of the estrous cycle using stained vaginal smears. Toxicol. Pathol..

[27] Andersen CL, Jensen JL, Orntoft TF (2004). Normalization of real-time quantitative reverse transcription-PCR data: a model-based variance estimation approach to identify genes suited for normalization, applied to bladder and colon cancer data sets. Cancer Res..

[28] Livak KJ, Schmittgen TD (2001). Analysis of relative gene expression data using real-time quantitative PCR and the 2(-delta delta C(T)) method. Methods (San Diego, CA).

[29] Schloss PD (2009). Introducing mothur: open-source, platform-independent, community-supported software for describing and comparing microbial communities. Appl. Environ. Microbiol.

[30] Kozich JJ, Westcott SL, Baxter NT, Highlander SK, Schloss PD (2013). Development of a dual-index sequencing strategy and curation pipeline for analyzing amplicon sequence data on the MiSeq Illumina sequencing platform. Appl. Environ. Microbiol..

[31] Zakrzewski M (2017). Calypso: a user-friendly web-server for mining and visualizing microbiome-environment interactions. Bioinformatics (Oxf., Engl.).

[32] McMurdie PJ, Holmes S (2013). phyloseq: an R package for reproducible interactive analysis and graphics of microbiome census data. PLoS ONE.

[33] Love MI, Huber W, Anders S (2014). Moderated estimation of fold change and dispersion for RNA-seq data with DESeq2. Genome Biol..

[34] Pruesse E, Peplies J, Glöckner FO (2012). SINA: accurate high-throughput multiple sequence alignment of ribosomal RNA genes. Bioinformatics.

[35] Clarke KR (1993). Non-parametric multivariate analyses of changes in community structure. Aust. J. Ecol..

[36] Douglas, G. M. et al. PICRUSt2: an improved and extensible approach for metagenome inference. *bioRxiv* 672295 (2019).

[37] Martire SI, Maniam J, South T, Holmes N, Westbrook RF, Morris MJ (2014). Extended exposure to a palatable cafeteria diet alters gene expression in brain regions implicated in reward, and withdrawal from this diet alters gene expression in brain regions associated with stress. Behav. Brain Res..

[38] Barker GR, Bird F, Alexander V, Warburton EC (2007). Recognition memory for objects, place, and temporal order: a disconnection analysis of the role of the medial prefrontal cortex and perirhinal cortex. J. Neurosci..

[39] Barker GR, Warburton EC (2011). When is the hippocampus involved in recognition memory?. J. Neurosci..

[40] Wen L (2008). Innate immunity and intestinal microbiota in the development of Type 1 diabetes. Nature.

[41] Reichelt AC, Morris MJ, Westbrook RF (2016). Daily access to sucrose impairs aspects of spatial memory tasks reliant on pattern separation and neural proliferation in rats. Learn Mem..

[42] Kim H (2016). Caloric restriction improves diabetes-induced cognitive deficits by attenuating neurogranin-associated calcium signaling in high-fat diet-fed mice. J. Cereb. Blood Flow. Metab..

[43] Li L, Wang Z, Zuo Z (2013). Chronic intermittent fasting improves cognitive functions and brain structures in mice. PLoS ONE.

[44] Belegri E, Eggels L, Unmehopa UA, Mul JD, Boelen A, la Fleur SE (2018). The effects of overnight nutrient intake on hypothalamic inflammation in a free-choice diet-induced obesity rat model. Appetite.

[45] Almeida-Suhett CP, Graham A, Chen Y, Deuster P (2017). Behavioral changes in male mice fed a high-fat diet are associated with IL-1β expression in specific brain regions. Physiol. Behav..

[46] Bove RM, Gerweck AV, Mancuso SM, Bredella MA, Sherman JC, Miller KK (2016). Association between adiposity and cognitive function in young men: Hormonal mechanisms. Obesity (Silver Spring).

[47] Gonzales MM (2014). Central adiposity and the functional magnetic resonance imaging response to cognitive challenge. Int. J. Obes..

[48] Spyridaki EC (2014). The association between obesity and fluid intelligence impairment is mediated by chronic low-grade inflammation. Br. J. Nutr..

[49] Thaiss CA (2016). Persistent microbiome alterations modulate the rate of post-dieting weight regain. Nature.

[50] Ravussin Y (2012). Responses of gut microbiota to diet composition and weight loss in lean and obese mice. Obesity (Silver Spring).

[51] Bhagavata Srinivasan SP, Raipuria M, Bahari H, Kaakoush NO, Morris MJ (2018). Impacts of diet and exercise on maternal gut microbiota are transferred to offspring. Front. Endocrinol..

[52] Cotillard A (2013). Dietary intervention impact on gut microbial gene richness. Nature.

[53] Turnbaugh PJ, Bäckhed F, Fulton L, Gordon JI (2008). Diet-induced obesity is linked to marked but reversible alterations in the mouse distal gut microbiome. Cell Host Microbe.

[54] Shiraev T, Chen H, Morris MJ (2009). Differential effects of restricted versus unlimited high-fat feeding in rats on fat mass, plasma hormones and brain appetite regulators. J. Neuroendocrinol..

[55] Papachristou E (2015). The relationships between body composition characteristics and cognitive functioning in a population-based sample of older British men. BMC Geriatr..

[56] Chen J-M (2014). Cognitive impairment among elderly individuals in Shanghai Suburb, China: association of C-reactive protein and its interactions with other relevant factors. Am. J. Alzheimers Dis. Other Dem..

[57] Erion JR (2014). Obesity elicits interleukin 1-mediated deficits in hippocampal synaptic plasticity. J. Neurosci..

[58] Noh HM (2017). Relationships between cognitive function and body composition among community-dwelling older adults: a cross-sectional study. BMC Geriatr..

[59] Narimani, M., Esmaeilzadeh, S., Azevedo, L. B., Moradi, A., Heidari, B., Kashfi-Moghadam, M. Association between weight status and executive function in young adults. *Medicina (Kaunas)* **55**, 363 (2019). 10.3390/medicina55070363 PMC6681338 31295973

[60] Golden, R. et al. Lean body mass, but not fat mass, is associated with hippocampal memory performance. *Curr. Dev. Nutr*. **3**, P14-011-19 (2019).

[61] Lagkouvardos I (2016). The Mouse Intestinal Bacterial Collection (miBC) provides host-specific insight into cultured diversity and functional potential of the gut microbiota. Nat. Microbiol..

[62] Dobranowski, P. A., Tang, C., Sauvé, J. P., Menzies, S. C., Sly, L. M. Compositional changes to the ileal microbiome precede the onset of spontaneous ileitis in SHIP deficient mice. *Gut Microbes* **10**, 578–598 (2019). 10.1080/19490976.2018.1560767 PMC6748580 30760087

[63] Chakraborty TR, Donthireddy L, Adhikary D, Chakraborty S (2016). Long-term high fat diet has a profound effect on body weight, hormone levels, and estrous cycle in mice. Med. Sci. Monit..

[64] Cabello E (2015). Effects of resveratrol on ovarian response to controlled ovarian hyperstimulation in ob/ob mice. Fertil. Steril..

[65] Hou YJ, Zhu CC, Duan X, Liu HL, Wang Q, Sun SC (2016). Both diet and gene mutation induced obesity affect oocyte quality in mice. Sci. Rep..

