# Supplementary material for: Intermittent cafeteria diet identifies fecal microbiome changes as a predictor of spatial recognition memory impairment in female rats
Source: Transl Psychiatry. 2020 Jan 27;10:36. doi: 10.1038/s41398-020-0734-9 (PMC7026185; doi:10.1038/s41398-020-0734-9)
Supplement: Supplementary file 1 — Supplementary Figures and Tables [file 41398_2020_734_MOESM1_ESM.docx]

**Supplementary Figures and Tables**

**Supplementary Table 1.** Taq assay probe information

| Gene name (abbreviation) | Unique assay identifier |
| --- | --- |
| Allograft inflammatory factor 1 (*Aif1*) | Rn00574125_g1 |
| Brain-derived neurotrophic factor (*Bdnf*) | Rn02531967_s1 |
| Claudin-5 (*Cldn5*) | Rn01753146_s1 |
| Dopamine receptor D1 (*Drd1*) | Rn03062203_s1 |
| Dopamine receptor D2 (*Drd2*) | Rn01418275_m1 |
| Glial fibrillary acidic protein (*Gfap*) | Rn01253033_m1 |
| Glucose transporter 1 (*Glut1*) | Rn01417099_m1 |
| Glucose transporter 3 (*Glut3*) | Rn00567331_m1 |
| Growth hormone secretagogue receptor (*Ghsr*) | Rn_00821417_m1 |
| Hypoxanthine phosephoribosyltransferase 1 (*Hprt1*) | Rn01527840_m1 |
| Inhibitor of nuclear factor kappa B kinase subunit beta (*Ikbkb*) | Rn00584379_m1 |
| Insulin receptor (*Insr*) | Rn00690703_m1 |
| Interleukin-1 beta (*Il1B*) | Rn00580432_m1 |
| Interleukin-6 (*Il6*) | Rn01410330_m1 |
| Leptin receptor (*Lepr*) | Rn01433205_m1 |
| Mammalian target of rapamycin (*Mtor*) | Rn00693900_m1 |
| Melanocortin 4 receptor (*Mc4r*) | Rn01491866_s1 |
| Occludin (*Ocln*) | Rn00580064_m1 |
| Serotonin receptor 1a (*Htr1a*) | Rn00561409_s1 |
| Synapsin 1 (*Syn1*) | Rn00569468_m1 |
| Toll-like receptor 2 (*Tlr2*) | Rn02133647_s1 |
| Toll-like receptor 4 (*Tlr4*) | Rn99999017_m1 |
| Tumour necrosis factor alpha (*Tnf*) | Rn99999017_m1 |
| Tyrosine 3-monooxygenase/tryptophan 5-monooxygenase activation protein zeta (*Ywhaz*) | Rn00755072_m1 |

Taq assays and their unique identifiers assessed in dorsal hippocampus.


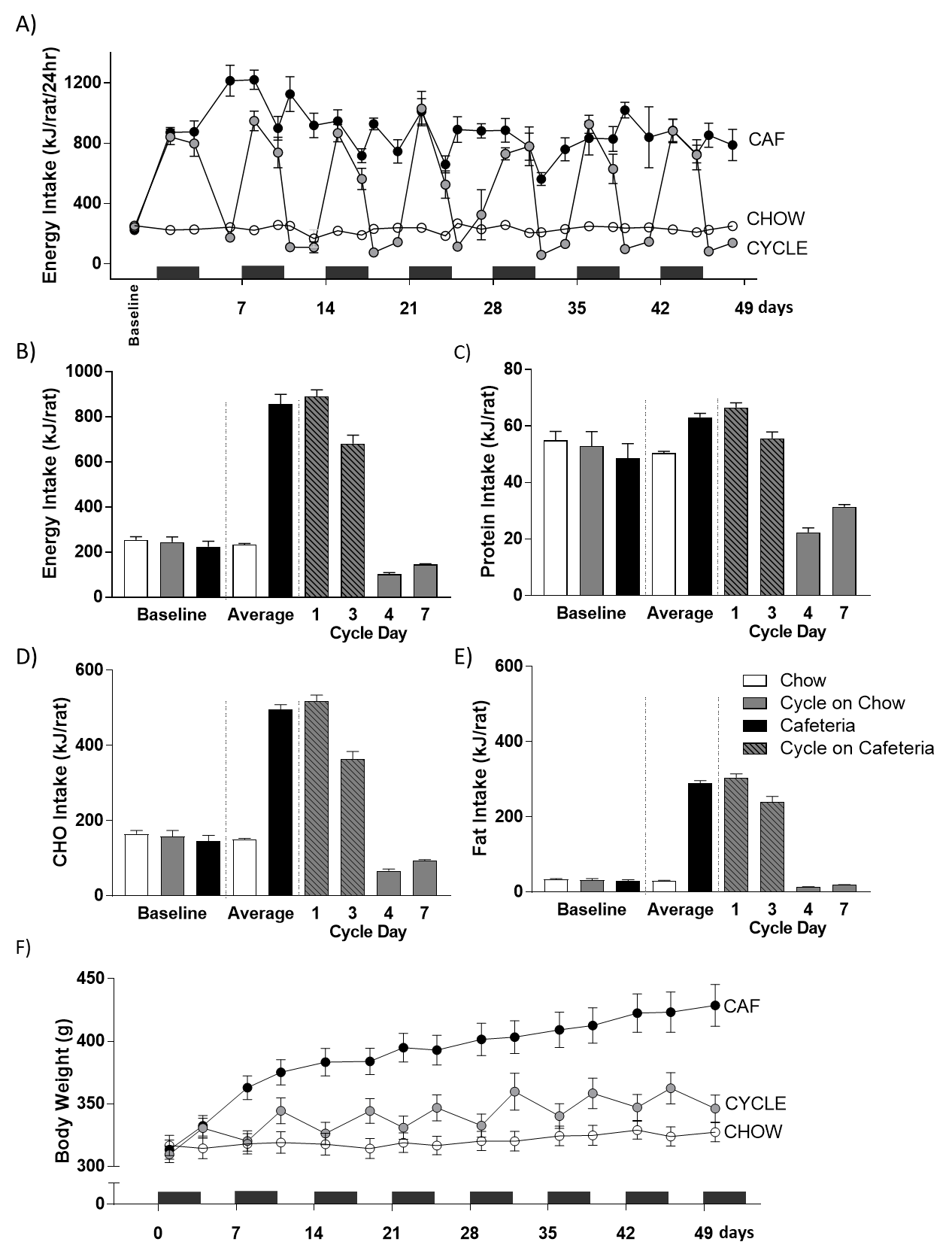


**Supplementary Figure 1.** Food intake and body weight gain across the study. (A) Average 24-hour energy intake over the study. (B) 24-hour total energy, (C) protein, (D) carbohydrate (CHO) and (E) fat intake, averaged across the study for Chow and Cafeteria groups, and averaged across the weekly cycle for Cycle rats where the first and last days of cafeteria diet exposure (Cycle days 1 and 3 respectively), and the first and last days of healthy chow diet exposure (Cycle days 4 and 7 respectively). Baseline measures were undertaken the week prior to any dietary exposure. Data expressed as mean ± SEM; n=4 cages. (F) Body weight over the study. Data expressed as mean ± SEM; n=12. 24-hour food intake measure and body weight data presented over time: black bars along x-axis represent when the Cycle group were consuming cafeteria diet.


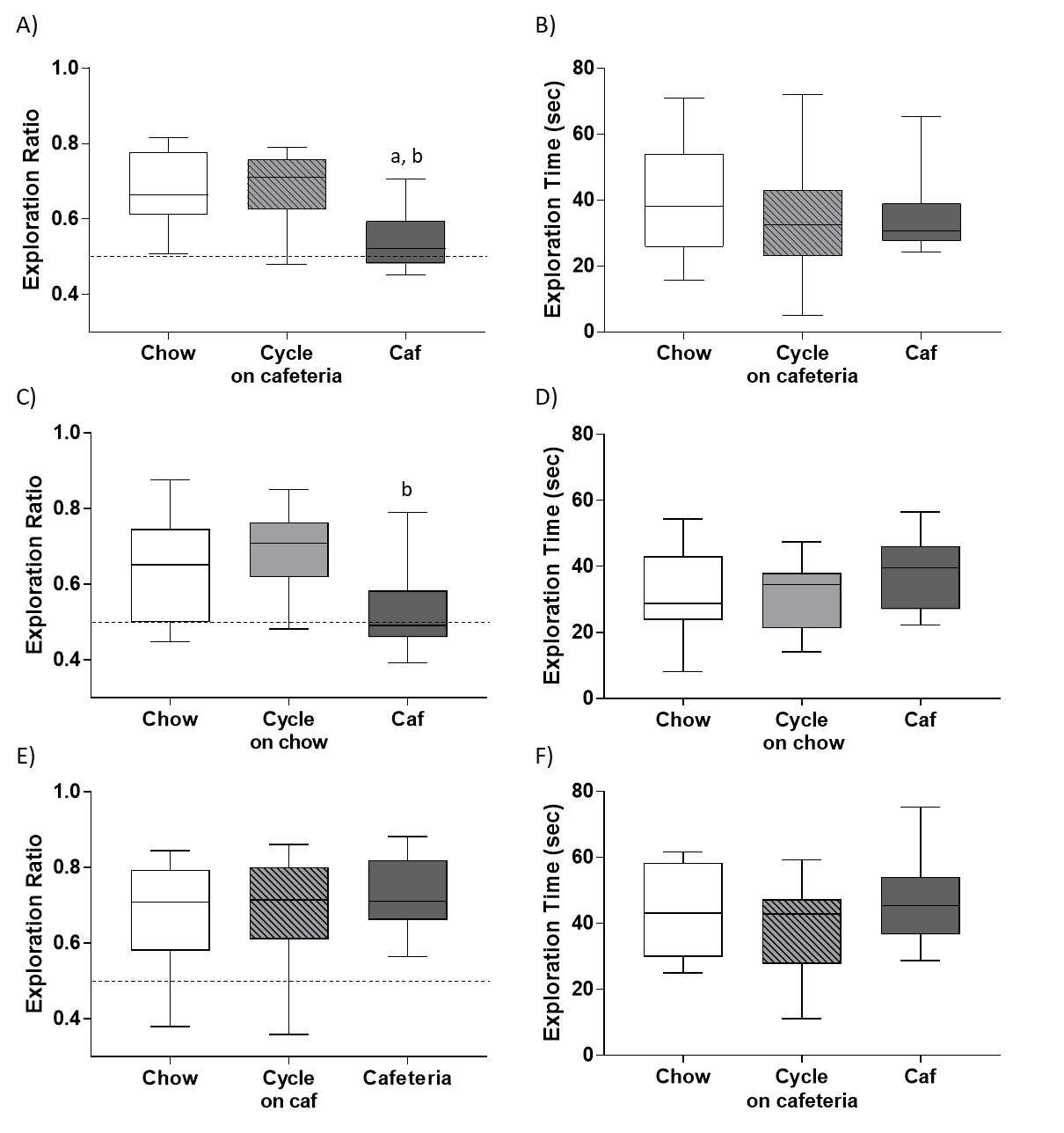


**Supplementary Figure 2.** Novel place recognition (A-D) and novel object recognition (E-F) performance over the experiment. (A) Place exploration ratio and (B) exploration time following 3 weeks of diet while Cycle rats were consuming cafeteria diet. (C) Place exploration ratio and (D) exploration time following 6 weeks of diet while Cycle rats were consuming healthy chow. (E) Object exploration ratio and (F) exploration time following 4 weeks of diet while Cycle rats were consuming cafeteria diet. Data are expressed as box-and-whisker plots (min, IQR, max); n=10-12; data were analyzed by one-way ANOVA followed by Tukey-adjusted post-hoc comparisons (^a^p<0.05 relative to Chow, ^b^p<0.005 relative to Cycle).


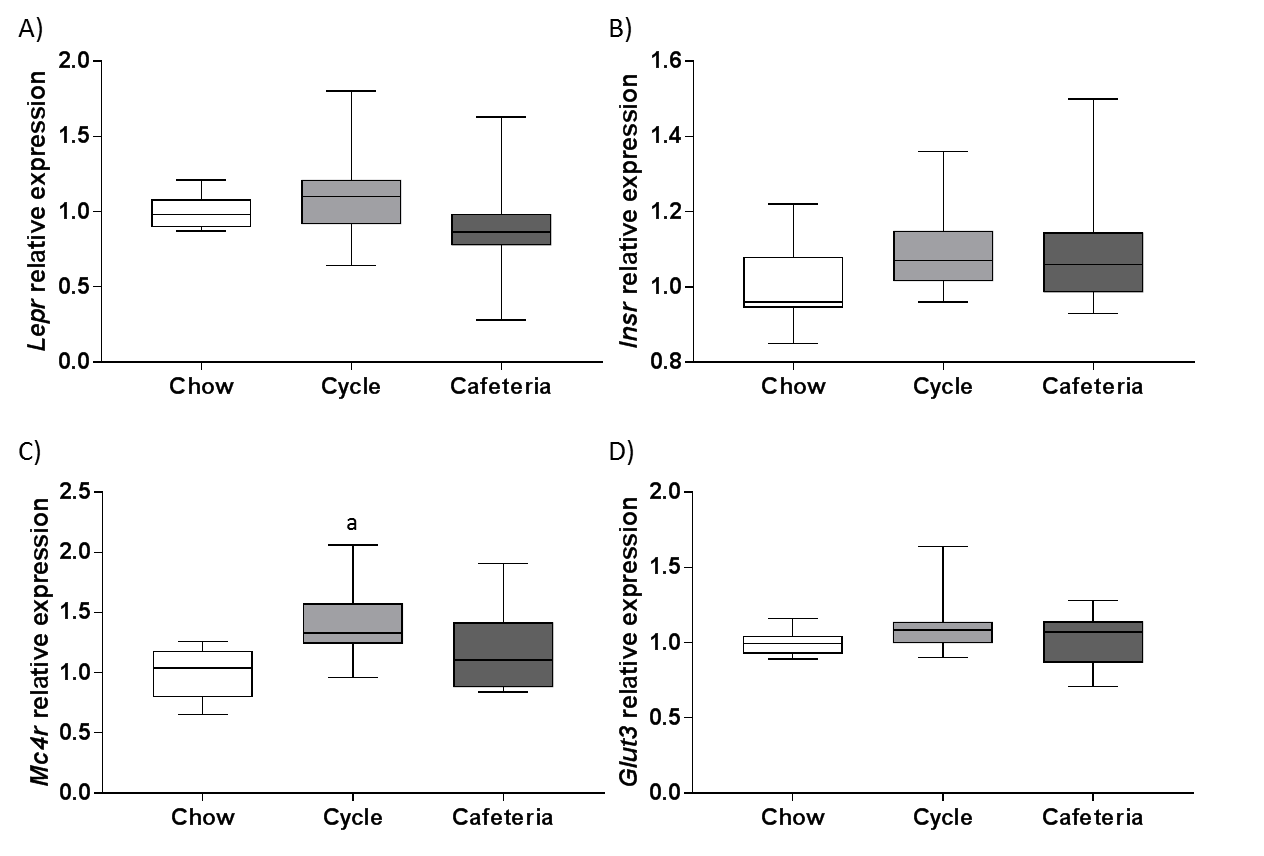


**Supplementary Figure 3.** Intermittent cafeteria diet exposure increased dorsal hippocampal Mc4r gene expression without affecting other metabolism-related genes. (A) *Lepr* expression. (B) *Insr* expression. (C) *Mc4r* expression. (D) *Glut3* expression. Data are expressed as box-and-whisker plots (min, IQR, max); n=10-12; data were analyzed by one-way ANOVA followed by Tukey-adjusted post-hoc comparisons (^a^p<0.05 relative to Chow). *Glut3*: glucose transporter 1, *Insr*: insulin receptor, *Lepr*: leptin receptor, *Mc4r*: melanocortin 4 receptor.

**Supplementary Table 2.** Correlations between variables of interest, place task performance at 6 weeks and *Coprobacter*_OTU66

|  | Place task performance | | *Coprobacter*_OTU66 | |
| --- | --- | --- | --- | --- |
| Variable | r | P-Value | r | P-value |
| Place task performance | 1 |  | -0.456 | **0.008** |
| Girth | -0.463 | **0.008** | 0.346 | **0.045** |
| Fat mass (percentage body weight) | -0.402 | **0.020** | 0.502 | **0.002** |
| Lean mass | -0.362 | **0.038** | -0.043 | 0.806 |
| Liver mass | -0.408 | **0.018** | 0.190 | 0.274 |
| Heart mass | -0.377 | **0.031** | 0.347 | **0.041** |
| Plasma leptin concentration | -0.424 | **0.018** | 0.331 | 0.060 |
| Plasma triglyceride concentration | -0.354 | **0.043** | 0.315 | 0.070 |
| Hippocampal *Aif1* | -0.483 | **0.007** | 0.426 | **0.015** |
| Hippocampal *Tlr2* | -0.457 | **0.011** | 0.452 | **0.009** |
| Hippocampal *Tlr4* | -0.288 | 0.123 | 0.366 | **0.040** |
| Hippocampal *Gfap* | -0.336 | 0.070 | 0.373 | **0.035** |
| *Coprobacter*_OTU66 | -0.456 | **0.008** | 1 |  |

Pearson correlations were performed to identify variables of interest significantly associated with place task performance at 6 weeks and relative abundance of Coprobacter_OTU66; N=34-35.
